# Physical activity, metabolic health and well-being in community-dwelling older adults: a scoping review

**DOI:** 10.3389/fmed.2026.1969460

**Published:** 2026-10-16

**Authors:** Fatih Sarıkaya, Oğuzhan Gül, Turhan Toros, Tülay Ceylan, Meryem Güler Yılmaz, Hatice Arslan, Mehmet Emre Eryücel, Mustafa Bahar

**Affiliations:** 1 Ministry of Youth and Sports, Adıyaman Provincial Directorate of Youth and Sports, Adıyaman, Türkiye; 2 Department of Health, Culture and Sports, Sivas Cumhuriyet University, Sivas, Türkiye; 3 Department of Coaching Education, Mersin University, Mersin, Türkiye; 4 Çorum Provincial Directorate of National Education, Ministry of National Education, Çorum, Türkiye; 5 Department of Recreation, Faculty of Sports Sciences, İstanbul Topkapı University, İstanbul, Türkiye; 6 Independent Researcher, Mersin, Türkiye; 7 Department of Sports Management, Faculty of Sports Sciences, Akdeniz University, Antalya, Türkiye; 8 Mersin Nutrition and Dietetics Clinic, Mersin, Türkiye

**Keywords:** 24-hour movement composition, ageing, cardiometabolic risk, cognition, compositional data analysis, sedentary behavior, sleep

## Abstract

**Background/objectives:**

Physical activity, sedentary time and sleep make up a closed 24-hour composition; reallocating time from one behavior to another has consequences for the metabolic profile and the well-being of the older adult. No single account has mapped which behaviors have been studied against which outcomes in this population. We conducted a scoping review to chart the exposures against the outcomes, summarize the associations, compare the assessment methods, and identify the gaps.

**Methods:**

Four databases (PubMed, Scopus, Web of Science and SPORTDiscus) were searched through May 2026, in line with the Arksey and O'Malley framework and PRISMA-ScR. Studies were eligible when they enrolled community-dwelling older adults with a mean age of 60 or above and reported a movement-behavior exposure against a metabolic or a well-being outcome. Database searches identified 21,995 records; 2,468 remained after de-duplication for title-and-abstract screening. Reference-list hand-searching yielded 193 additional citations, three of which proceeded to full-text assessment. Overall, 82 full-text reports were assessed for eligibility and 27 studies entered the charted evidence base.

**Results:**

Cognition was the dominant well-being outcome; more activity and less sitting went with better cognitive performance across several cohorts. In one cross-sectional study of short-sleeping older adults, the cognitive benefit appeared to plateau at approximately 800–960 MET-minutes per week, although no other included study tested that threshold. The metabolic signal was real but thinner, and it rested largely on cross-sectional compositional work. Quality of life was rarely measured.

**Conclusions:**

The daily movement composition tracks with both the cardiometabolic and the well-being outcomes. Encouraging the older adult to move more, sit less and sleep adequately is supported by the direction of the evidence. What the field still lacks are the causal design, the quality-of-life outcome, and investigations that isolate activity from the surrounding lifestyle.

## 1 Introduction

Populations across the globe have been ageing at a pace without precedent, and the later decades are set to carry a metabolic drift that gathers quietly. With advancing age, the sensitivity of the tissues to insulin falls, the visceral and the ectopic fat enlarge (1), and the skeletal muscle is lost in both quantity and quality, a cluster that, alongside the insulin resistance of later life, prepares the ground for type 2 diabetes, the metabolic syndrome and cardiovascular disease (2). Below the named markers, the metabolome itself shifts, and the shape of that shift is not fixed by the age alone. In a study of older women, the demographic and the lifestyle factors together left a distinct print on the circulating metabolites (3), while across the span from vigor to frailty in older men the metabolites tracked the decline (4). The muscle sits at the center of the picture, since the signals released through exercise, the exerkines, govern the mitochondrial homeostasis on which the metabolic health of the ageing muscle depends (5).

In this respect, lifestyle is not a backdrop but a determinant. In a cross-sectional and longitudinal study of 139,634 workers, the sociodemographic and the lifestyle factors carried the risk of the metabolic syndrome and of the hypertriglyceridemic-waist phenotype (6), and the guidance for older adults reflects that weight, advising at least 150–300 min of moderate activity in a week together with the muscle-strengthening and the balance work (7). The way the field interprets the activity has also shifted. In recent work, the day is now treated as a closed composition of the behaviors rather than as a single dose of exercise. The activity, the sedentary time and the sleep sum to 24 h, so that an addition to one of them means a subtraction from another.

Well-being is the other half of the account. General well-being in the later life, taken here to cover quality of life, mood, cognition, sleep and physical function, tends to follow the state of the body, and physical function is the hinge that joins the two halves. In the ENRGISE exercise trial, the metabolomic signatures marked the physical function and its trajectory in older adults (8). So, the metabolic and the well-being threads are not separate stories but two readings of the same active life.

Although the evidence has grown, it sits scattered across the exposures, the outcomes and the designs, and no single account has mapped, for healthy community-dwelling older adults, which movement behaviors have been studied against which metabolic and well-being outcomes. Earlier reviews have addressed parts of the picture. Systematic reviews and meta-analyses have examined the association of sedentary behavior with cardiometabolic risk and functional outcomes in older adults (9), while separate reviews have synthesized the cognitive effects of physical activity across the lifespan or within specific clinical populations. More recently, compositional-data approaches have been reviewed from a methodological standpoint (10), and narrative accounts have situated sleep alongside activity within the 24-hour framework. What remains absent, however, is a scoping review that charts all three movement behaviors against both the metabolic and the well-being outcomes simultaneously, within a single community-dwelling older-adult population frame. Our review was written to fill that gap. We map physical activity, sedentary time and sleep as predictors of the cardiometabolic markers and of well-being in community-dwelling older adults with a mean age of 60 or above. Four objectives give the work its order: to chart which exposures have been studied against which outcomes; to summarize the reported associations with the cardiometabolic markers as well as with the domains of well-being; to place the assessment and the follow-up methods of the community studies alongside one another; and to name the gaps, then turn the findings toward the design of future work and the refinement of the guidelines. Figure 1 presents the 24-hour movement composition as a closed daily budget of 1,440 minutes, partitioned among sleep, sedentary time, light activity and moderate-to-vigorous activity.

**Figure 1:**
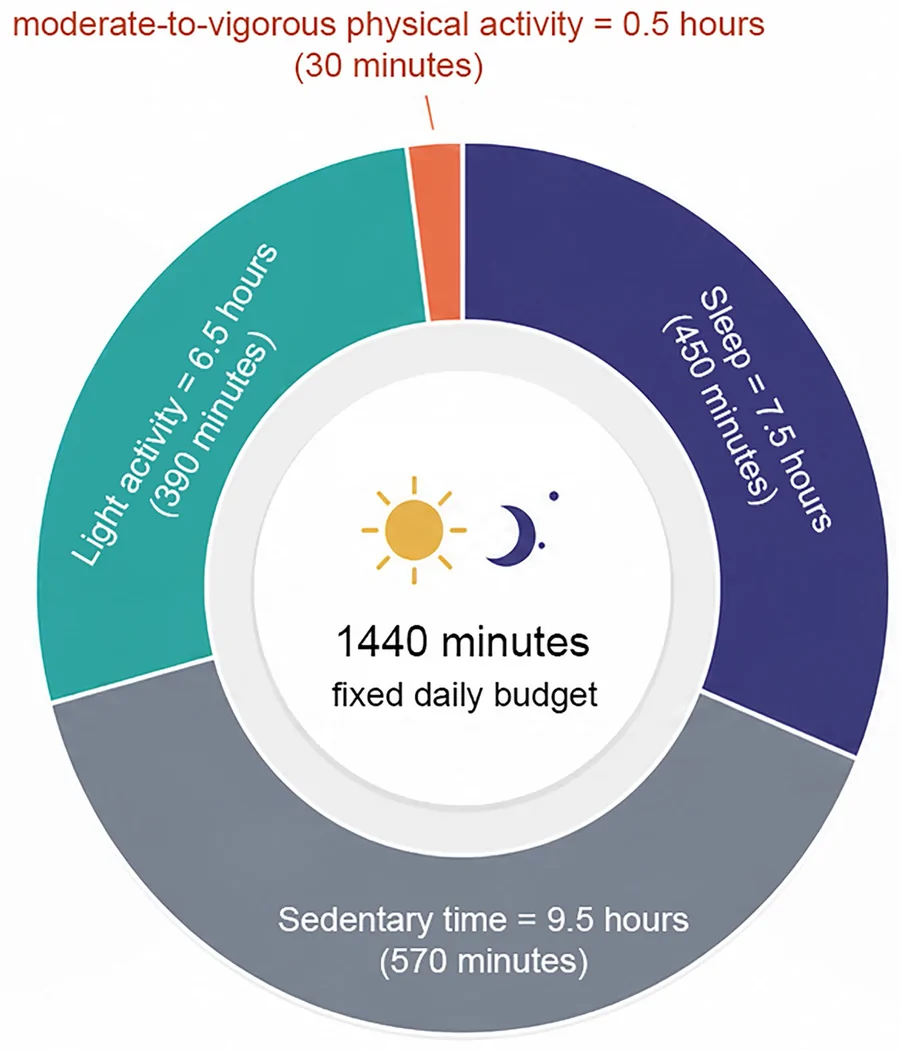
The 24-hour movement composition in community-dwelling older adults. The day is a fixed budget of 1,440 minutes, distributed among four movement behaviors: sleep (450 min, 31.3%), sedentary time (570 min, 39.6%), light-intensity activity (390 min, 27.1%) and moderate-to-vigorous physical activity (30 min, 2.1%). Proportions are drawn from a representative compositional sample of older adults aged 68 years (14). Because the four components sum to the whole day, time reallocated to one behavior is necessarily taken from the others.

## 2 Methods

### 2.1 Study design and reporting

We conducted a scoping review following the methodological framework of Arksey and O'Malley (11), as refined by Levac et al. (12), and reported the review in accordance with the PRISMA extension for Scoping Reviews (PRISMA-ScR) (13). The review was designed to map and synthesize the available evidence linking physical activity, sedentary behavior, sleep, and related 24-hour movement patterns with cardiometabolic health and well-being in community-dwelling older adults.

### 2.2 Review question and PICOS framework

The review question was formulated to determine how physical activity, sedentary behavior, sleep, and related 24-hour movement patterns are associated with cardiometabolic health and well-being among community-dwelling older adults. In accordance with the journal's requirements, the eligibility framework was additionally structured according to the Population, Intervention/Exposure, Comparator, Outcomes, and Study design (PICOS) components.

### 2.3 Participants

The population comprised community-dwelling older adults with a mean age of 60 years or older. For each potentially eligible study, the mean age was verified from the demographic table or participant-characteristics section of the full-text report rather than from the abstract alone, because several abstracts reported only an age range without a sample mean.

### 2.4 Interventions/exposures

The interventions or exposures of interest included habitual or prescribed physical activity and exercise, sedentary behavior, sleep, and 24-hour movement-behavior compositions or modeled reallocations.

### 2.5 Comparators

Comparators included alternative levels or patterns of movement behavior, different exercise doses or conditions, control conditions, or modeled reallocations of time between movement behaviors, where applicable. Studies without an explicit comparator were also eligible when their observational design addressed the review objectives.

### 2.6 Outcomes

Outcomes included cardiometabolic indicators, such as glucose regulation, insulin-related measures, lipid profiles, blood pressure, body composition, inflammatory markers, and other relevant metabolic outcomes, together with well-being outcomes including cognition, mood, sleep-related outcomes, physical function, and quality of life.

### 2.7 Study designs and eligibility criteria

Studies were eligible if they were published from January 2010 to the date of the final search in May 2026, enrolled community-dwelling older adults with a mean age of 60 years or above, and reported a movement-behavior exposure (physical activity, sedentary time, or sleep) in relation to a metabolic or well-being outcome. Well-being was defined broadly to include quality of life, mood, cognition, sleep-related outcomes, and physical function. Because these subdomains are conceptually distinct and because sleep serves both as an exposure (a component of the 24-hour movement composition) and as an outcome (sleep quality, variability, and efficiency), the subdomains are reported separately in the synthesized findings rather than combined into a single well-being index. We included primary studies with observational or interventional designs published in English. The 2010 lower date limit was applied to focus the review on contemporary evidence relevant to current movement-behavior assessment and analytical approaches. We excluded samples recruited on the basis of a defining metabolic or other disease at baseline, samples below the age threshold, records without a relevant exposure or outcome, and reviews, protocols, and conference abstracts. For every included study, the mean sample age was confirmed from the full-text demographic table rather than from the abstract alone. Where an abstract reported only an age range (e.g., 60–89 years), the full-text mean was retrieved and verified against the ≥60-year criterion. All 27 included studies met the threshold on the basis of a reported mean ± SD; no proxy rule was required.

### 2.8 Review protocol and registration

No review protocol was prospectively registered before data extraction commenced. The review was conducted according to the methodological and reporting frameworks described above.

### 2.9 Data sources

PubMed, Scopus, Web of Science Core Collection, and SPORTDiscus were searched in May 2026. Reference lists of relevant reports were also hand-searched to identify additional potentially eligible studies.

### 2.10 Search strategy

Database-specific search strategies combined population terms referring to older and community-dwelling adults with movement-behavior and health-related terms; where applicable, study-design terms and database-specific filters were also used. Complete search strings, field codes, limits, filters, and database-specific result counts are reported in Supplementary Table S1. The searches identified 21,995 records across the four databases before de-duplication. In addition, reference lists of relevant reports were hand-searched, yielding 193 citations for supplementary screening. Of these, three potentially eligible reports were identified for full-text assessment.

### 2.11 Study selection and data extraction

Records from the four databases were imported into Zotero reference management software and de-duplicated in two stages: an automated pass based on DOI, title, and author-name matching, followed by a manual review of flagged near-duplicates. Consolidating and de-duplicating the records left 2,468 records for screening after 19,527 duplicates had been removed. The duplicate proportion (89%) is high relative to the 40%–65% range typical of four-database reviews, but it is consistent with the near-complete indexing overlap among Scopus, Web of Science, and PubMed for English-language biomedical and sport-science journals published after 2010. Because the search strategies were intentionally broad and used comparable controlled-vocabulary and free-text terms across all four databases, the same records were retrieved repeatedly; the resulting inflation of the pre-deduplication total reflects retrieval overlap rather than an overly aggressive deduplication step. Title-and-abstract screening, full-text eligibility assessment, and data charting were sequentially reviewed by four authors (FS, OG, TT, and TC). At each stage, the records and eligibility decisions were successively checked by the reviewers, and any differences in interpretation were discussed until consensus was reached. Title-and-abstract screening excluded 2,389 records, leaving 79 reports for full-text assessment. In parallel, 193 citations identified through hand-searching of reference lists underwent supplementary citation-level screening; 190 did not proceed to full-text retrieval, while three potentially eligible reports were retrieved and assessed in full text. These three reports, together with the 79 database-derived reports, resulted in 82 full-text reports assessed for eligibility. Twenty-seven of these reports entered the charted synthesis; of the remaining 55, 16 were retained as clinical or contextual references for the Discussion and 39 were excluded from the charted evidence base.

Data from eligible studies were charted using a standardized form that recorded the author and year, country and study design, sample size and mean age, exposure and its measurement and dose where applicable, metabolic and well-being outcomes, and the reported direction of effect. The charted data were sequentially checked by FS, OG, TT, and TC, and discrepancies were resolved through discussion and consensus. The charting form was piloted on an initial subset of studies and refined before full data extraction. Figure 2 presents the flow of records through the review.

**Figure 2:**
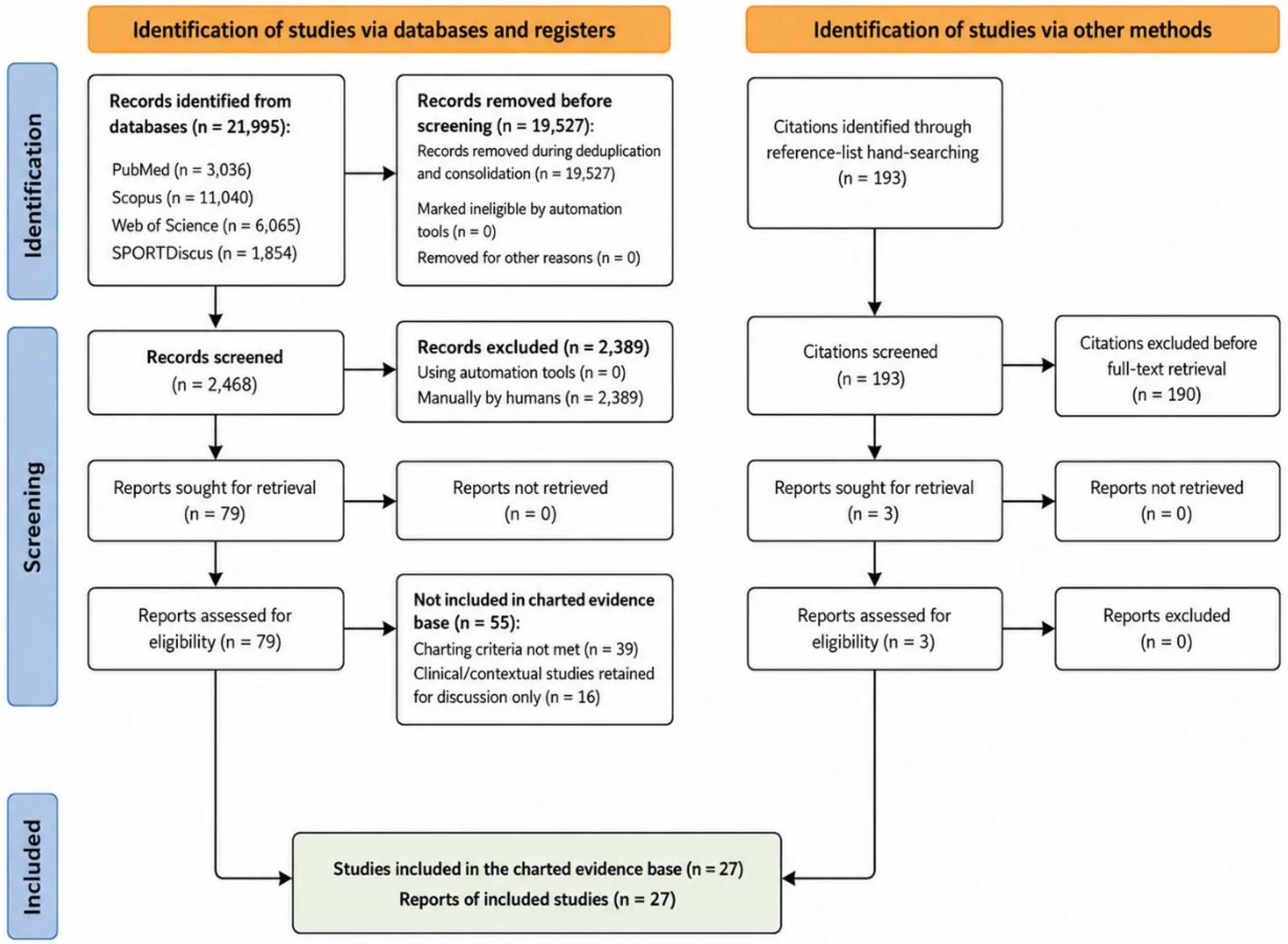
Flow diagram detailing the identification of records (n) and the selection of included studies. No records were excluded through automation tools; all screening was performed manually at every stage. Adapted from the PRISMA 2020 flow-diagram template (53).

### 2.12 Risk of bias assessment

Risk of bias was assessed according to study design using established design-specific appraisal tools. Analytical cross-sectional studies were evaluated using the revised JBI critical appraisal tool for analytical cross-sectional studies, and cohort studies were assessed using the revised JBI tool for cohort studies. Randomized controlled trials were evaluated using the JBI critical appraisal tool for randomized controlled trials, whereas non-randomized intervention studies, where applicable, were evaluated using the JBI tool for quasi-experimental studies. For randomized crossover trials, the Cochrane Risk of Bias 2 (RoB 2) tool for crossover trials was applied to account for design-specific concerns, including period and carryover effects.

Because the JBI checklists and the Cochrane RoB 2 tool use different response formats, individual checklist responses were converted to a common three-tier overall rating as follows. For JBI-appraised studies, a rating of “low risk” was assigned when all applicable checklist items received a “yes” response; “some concerns” was assigned when one or more items were answered “no” or “unclear” but the identified limitations were judged unlikely to materially alter the study's main findings; and “high risk” was assigned when two or more items indicated methodological shortcomings considered likely to affect the validity of the results. For the single crossover trial appraised with RoB 2, the tool's own algorithmic overall judgment was applied directly. Risk-of-bias assessments were used to contextualize the interpretation of the evidence and were not used as criteria for study exclusion, consistent with the mapping purpose of the scoping review. Detailed study-level assessments are presented in Supplementary Table S3.

### 2.13 Data analysis

The charted studies were synthesized narratively, organized around the 24-hour movement composition and the two outcome domains. Where full-text data were available, effect estimates were extracted and reported in their original units; otherwise, the direction of the association was recorded. No meta-analysis was undertaken because the study designs, exposures, and outcome measures were considered too heterogeneous for meaningful quantitative pooling.

## 3 Results

### 3.1 Study selection and characteristics

Database searches identified 21,995 records. After removal of 19,527 duplicates, 2,468 records underwent title-and-abstract screening; 2,389 were excluded and 79 proceeded to full-text assessment. Reference-list hand-searching yielded 193 additional citations, of which 190 did not proceed to full-text retrieval and three were assessed in full text. In total, 82 full-text reports were assessed for eligibility. Twenty-seven studies were included in the charted evidence base; 39 reports did not meet the charting criteria and 16 were retained solely as clinical or contextual references for the Discussion. Detailed reasons for non-inclusion are provided in Supplementary Table S2.

The characteristics of the 27 included studies are summarized in Table 1. Of these, 12 reported at least one cardiometabolic outcome (Table 2), 22 reported at least one well-being outcome (Table 3), and seven contributed outcomes to both domains. Study designs ranged from acute randomized crossover trials to prospective cohort studies with follow-up extending to ten years, although analytical cross-sectional designs predominated. The included studies examined habitual and prescribed physical activity, sedentary behavior, sleep, and broader 24-hour movement patterns using heterogeneous measurement approaches. Accordingly, the synthesized findings were organized around the 24-hour movement composition and the cardiometabolic and well-being outcome domains.

**Table 1:** Characteristics of the 27 included studies.

Study	Design	N	Mean age	Exposure(s) measured	Measurement
Anastasiou et al. (2018) (30)	Cross-sectional	1,716	≥65 y	Mediterranean lifestyle index including PA, sleep, diet, social/intellectual aspects	NR
Breneman et al. (2019) (23)	RCT/trial	49	64.53 ± 3.83 y	Moderate-intensity walking, low vs. high dose	Prescribed (trial)
Dong et al. (2026) (5)	Cross-sectional	373	71.0 ± 5.4 y	Physical activity collected as predictor/covariate	NR
Dumuid et al. (2018b) (16)	Cross-sec (CoDA)	NR	65 ± 3 y	MVPA, sedentary behavior, sleep/time-use behaviors	NR
Dzierzewski et al. (2014) (34)	Longitudinal analysis	79	63.58 ± 8.66 y	Exercise behavior/daily activity	Self-report; trial
Edwards and Loprinzi (2017) (24)	Cross-sectional	2,472	60–85 y	Sedentary behavior and MVPA	Self-report
Fanning et al. (2017) (26)	Cross-sec (ISM)	247	65.4 ± 4.6 y	Sedentary behavior, LPA, MVPA, sleep by accelerometer	Device-based
Gennuso et al. (2013) (18)	Cross-sectional	1,914	≥65 y	Accelerometer-derived sedentary behavior and MVPA	Device-based
Ghasemi and Cheraghbirjandi (2024) (22)	RCT/trial	40	66.42 ± 4.15 y (exercise) 67.10 ± 3.92 y (control)	Multicomponent training: aerobic, resistance, and balance exercise	Prescribed (trial)
Hu et al. (2025) (29)	Cohort	5,381	≥65 y	Physical activity, diet, sleep, cognitive and social activities	NR
Liang et al. (2024) (14)	Cross-sec (CoDA)	4,562	67.68 ± 5.03 y	MVPA, LPA, sedentary behavior, sleep	NR
Maréchal et al. (2026) (20)	RCT (crossover)	12	72.3 ± 4.8 y	Light-intensity treadmill walking breaks	Prescribed (trial)
Mellow et al. (2022) (27)	Cross-sectional	384	65.5 ± 3.0 y	24-h time use by accelerometry; recreational PA; TV watching	Device-based
Nagayama et al. (2021) (21)	RCT/trial	26	72 ± 5 y	Self-selected increase in daily physical activity	Prescribed (trial)
Nijholt et al. (2016) (32)	Cross-sectional	2,165	55–85 y	Moderate-intensity PA by questionnaire	Self-report
Powell et al. (2020) (15)	Cross-sec (CoDA)	366	64.6 ± 5.3 y	Sleep, sedentary time, standing, LIPA, MVPA by activPAL	Device-based
Ryan et al. (2019) (17)	Cross-sec (CoDA)	93	60–89 y	Sleep, sedentary behavior, physical activity	NR
Scotti et al. (2026) (33)	Cross-sectional	204	72.9 ± 6.65 y	Lifelong physical activity; diet; sleep; cognitive reserve	NR
Sewell et al. (2023) (37)	RCT/trial	89	68.76 ± 5.32 y	Moderate- and high-intensity exercise	Prescribed (trial)
Shakersain et al. (2018) (31)	Cohort	2,223	≥60 y	Physical, mental, and social leisure activities with Nordic Prudent Dietary Pattern	NR
Sheffler et al. (2025) (36)	Cross-sectional	276 older sample; 843 MIDUS	55 + in older sample	Exercise/physical activity as lifestyle moderator; eating habits	NR
Smagula et al. (2022) (39)	Cross-sectional	Weighted to ∼32 million US older adults	≥65 y	24-h activity pattern phenotypes by accelerometry	Device-based
Walker et al. (2024) (19)	RCT/trial	66	72.9 ± 2.8 y (intervention) 72.2 ± 2.3 y (control)	Step reduction versus usual activity	Prescribed (trial)
Wei et al. (2021) (25)	Cross-sec (ISM)	3,086	≥60 y	Sedentary activity, walking/bicycling, MVPA; self-reported	Self-report
Wilckens et al. (2018) (35)	Cross-sectional	112 total; 53 older	Older subgroup 55–80 y	METs during waking time; accelerometer PA	Device-based
You et al. (2023) (28)	Cross-sectional	1615	>60 y	Physical exercise volume (MET-min/week)	Self-report
Zamora et al. (2025) (38)	Cross-sectional	2,812	≥65 y	MVPA, sedentary behavior, sleep guideline adherence	Self-report

BMI, body mass index; BP, blood pressure; CoDA, compositional data analysis; CRP, C-reactive protein; DSST, Digit Symbol Substitution Test; HDL, high-density lipoprotein; HR, hazard ratio; ISM, isotemporal substitution model; MCI, mild cognitive impairment; MET, metabolic equivalent; MVPA, moderate-to-vigorous physical activity; NEFA, non-esterified fatty acids; NR, not reported; PA, physical activity; PHQ-9, Patient Health Questionnaire-9; RER, respiratory exchange ratio; RMR, resting metabolic rate; SB, sedentary behavior; VO_2_peak, peak oxygen uptake; WASO, wake after sleep onset. The symbol∼denotes a reported association.

**Table 2:** Cardiometabolic outcomes (studies reporting a metabolic marker; *n* = 12).

Study	Design	Metabolic outcome(s)	Key finding
Breneman et al. (2019) (23)	RCT/trial	Cardiorespiratory fitness (VO_2_peak)	Higher cardiorespiratory fitness (VO_2_peak) related to steadier night-to-night sleep
Dong et al. (2026) (5)	Cross-sectional	Resting metabolic rate; body composition	RMR prediction equations; PA and sleep entered as covariates, not the main exposure
Dumuid et al. (2018b) (16)	Cross-sec (CoDA)	Cardiorespiratory fitness; obesity; cardiometabolic risk	Time-use composition∼cardiorespiratory fitness, adiposity, cardiometabolic risk
Gennuso et al. (2013) (18)	Cross-sectional	Weight; BMI; waist circumference; CRP; glucose	SB (mean 9.4 h/day)∼poorer weight, BMI, waist, CRP (all *p* < 0.01), glucose (*p* = 0.04), independent of MVPA
Ghasemi and Cheraghbirjandi (2024) (22)	RCT/trial	Adropin; endothelial function; insulin resistance	Multicomponent training raised adropin, NO, VO_2_max; lowered glucose, insulin, insulin resistance, BP
Liang et al. (2024) (14)	Cross-sec (CoDA)	Cardiometabolic health markers	MVPA and sleep∼better markers (all *P* < 0.001) except BP; light activity∼waist circ. (B = 0.313, *p* = 0.009)
Maréchal et al. (2026) (20)	RCT (crossover)	Metabolic flexibility; respiratory exchange ratio	Breaking up sitting shifted RER (*P* = 0.002) and NEFA (*p* = 0.041) acutely
Nagayama et al. (2021) (21)	RCT/trial	Postprandial triglycerides	More free-living activity did not lower postprandial triglycerides (1.78 vs. 1.31 mmol/L, *p* = 0.515)
Powell et al. (2020) (15)	Cross-sec (CoDA)	Body composition; blood lipids; glucose-control markers	Lean and fat mass fell with light activity, rose with standing (all *p* < 0.05); among blood markers only triglycerides moved
Ryan et al. (2019) (17)	Cross-sec (CoDA)	Endocrine cardiometabolic risk markers	Movement and sleep profiles differed between high- and low-cardiometabolic-risk groups
Sheffler et al. (2025) (36)	Cross-sectional	Multimorbidity/chronic health conditions	Sleep quality mediated the adversity to multimorbidity link; lifestyle moderators differed by sample
Walker et al. (2024) (19)	RCT/trial	Metabolic health markers	2-wk step reduction: HDL 1.62 to 1.55 mmol/L (*p* = 0.017), recovered after rehab; metabolic harm limited

BMI, body mass index; BP, blood pressure; CoDA, compositional data analysis; CRP, C-reactive protein; DSST, Digit Symbol Substitution Test; HDL, high-density lipoprotein; HR, hazard ratio; ISM, isotemporal substitution model; MCI, mild cognitive impairment; MET, metabolic equivalent; MVPA, moderate-to-vigorous physical activity; NEFA, non-esterified fatty acids; NR, not reported; PA, physical activity; PHQ-9, Patient Health Questionnaire-9; RER, respiratory exchange ratio; RMR, resting metabolic rate; SB, sedentary behavior; VO_2_peak, peak oxygen uptake; WASO, wake after sleep onset. The symbol∼denotes a reported association.

**Table 3:** Well-being outcomes (studies reporting cognition, sleep, mood or physical function; *n* = 22).

Study	Design	Well-being domain	Key finding
Anastasiou et al. (2018) (30)	Cross-sectional	Cognition	Higher Mediterranean-lifestyle index∼lower odds of MCI and low cognition
Breneman et al. (2019) (23)	RCT/trial	Sleep	Structured walking reduced night-to-night WASO variability; arose ∼9 min earlier after exercise (*p* = 0.01)
Dong et al. (2026) (5)	Cross-sectional	Sleep	Sleep duration measured alongside RMR as a covariate
Dzierzewski et al. (2014) (34)	Longitudinal analysis	Sleep	Exercise and sleep reciprocally related across chronic and acute time scales
Edwards and Loprinzi (2017) (24)	Cross-sectional	Cognition	SB ≥5 h/day∼lower DSST (β = −3.1; 95% CI −5.8 to −0.4; *p* = 0.02); attenuated to β = −2.5 (*p* = 0.07) with MVPA
Fanning et al. (2017) (26)	Cross-sec (ISM)	Cognition (executive)	Substituting sedentary time with active behaviors or sleep estimated for self-regulation and executive function
Gennuso et al. (2013) (18)	Cross-sectional	Physical function	Higher sedentary time∼more functional limitations (*p* < 0.01)
Ghasemi and Cheraghbirjandi (2024) (22)	RCT/trial	Sleep	Multicomponent training reduced sleep disorders
Hu et al. (2025) (29)	Cohort	Cognition	Healthiest lifestyle profile∼lower cognitive impairment risk (HR = 0.693; 95% CI 0.553–0.868); ∼7.5 y longer impairment-free
Liang et al. (2024) (14)	Cross-sec (CoDA)	Mental health; fitness	Better 24-h composition∼better mental health and physical fitness (*p* < 0.001)
Mellow et al. (2022) (27)	Cross-sectional	Cognition	24-h time-use composition and contextual behaviors∼cognitive performance
Nijholt et al. (2016) (32)	Cross-sectional	Cognition	Sufficient PA together with healthy diet∼better cognitive functioning
Scotti et al. (2026) (33)	Cross-sectional	Cognition (executive, memory)	Combined lifelong PA, diet and sleep∼better executive function
Sewell et al. (2023) (37)	RCT/trial	Sleep; cognition	Baseline sleep moderated exercise-induced cognitive change
Shakersain et al. (2018) (31)	Cohort	Cognition	Active lifestyle strengthened the diet to cognition link; slower cognitive decline
Sheffler et al. (2025) (36)	Cross-sectional	Sleep	Sleep quality mediated the adversity to multimorbidity link
Smagula et al. (2022) (39)	Cross-sectional	Mood (depression); cognition	Across four 24-h activity phenotypes depression rose 3.5 to 9.0% (*p* = 0.004); weak/disrupted rhythm∼worse cognition
Walker et al. (2024) (19)	RCT/trial	Physical function	Step reduction caused only limited function loss; strength recovered after rehab (*Δ*15%, *p* < 0.001)
Wei et al. (2021) (25)	Cross-sec (ISM)	Cognition	Replacing sedentary time with walking/cycling or MVPA∼better cognitive scores
Wilckens et al. (2018) (35)	Cross-sectional	Sleep; cognition	Sleep efficiency (not total sleep time) mediated PA to executive control, working memory, switching, recall
You et al. (2023) (28)	Cross-sectional	Cognition	Exercise∼better cognition up to ∼800–960 MET-min/wk in short-sleep subgroup (DSST β = 0.555 per 100 MET-min/wk; 95% CI 0.332–0.778; *p* < 0.001), then saturation
Zamora et al. (2025) (38)	Cross-sectional	Mood (depression)	Non-adherence to 24-h movement guidelines∼higher depression risk (PHQ-9)

BMI, body mass index; BP, blood pressure; CoDA, compositional data analysis; CRP, C-reactive protein; DSST, Digit Symbol Substitution Test; HDL, high-density lipoprotein; HR, hazard ratio; ISM, isotemporal substitution model; MCI, mild cognitive impairment; MET, metabolic equivalent; MVPA, moderate-to-vigorous physical activity; NEFA, non-esterified fatty acids; NR, not reported; PA, physical activity; PHQ-9, Patient Health Questionnaire-9; RER, respiratory exchange ratio; RMR, resting metabolic rate; SB, sedentary behavior; VO_2_peak, peak oxygen uptake; WASO, wake after sleep onset. The symbol∼denotes a reported association.

### 3.2 Synthesized findings

#### 3.2.1 The 24-hour movement composition

Four components made up the composition examined in the charted studies. Sleep occupied the largest share of the day and entered the analyses as both a duration and a quality. Sedentary behavior was defined as the waking time spent sitting or reclining at a very low cost in energy, at or below 1.5 metabolic equivalents of task (METs); it was distinguished from mere inactivity, since a person could satisfy an activity target and still sit for long unbroken stretches. Light-intensity activity covered the unhurried movement of the daily round: standing, strolling and household tasks. Moderate-to-vigorous activity stood at the upper end, encompassing brisk walking, cycling and structured exercise, and it was the part of the day that the earlier guidance enumerated first. Because the four components summed to the whole day, the studies interpreted them together, asking what became of the markers when 30 min were reallocated from the sedentary share to the active or the sleeping share.

For the most part, the activity in the charted set was habitual rather than prescribed. Participants were not placed on a program; instead, their free-living movement was observed, quantified in steps or in minutes, and related to the outcomes. In a smaller group of trials, the exercise was prescribed, and in those studies a defined modality reappeared: a multicomponent program combining aerobic, resistance, and balance work; a walking dose set at a moderate intensity; or a bout of light walking that interrupted prolonged sitting. Structured modalities had not vanished from this literature; they sat as the minority within a body of work concerned mostly with the free-living day.

How the day was quantified shaped what each study could establish. The device-based studies fixed an accelerometer or an activPAL on the body, together with actigraphy for the sleep, capturing the timing as well as the intensity but remaining blind to the kind of the activity. In the self-report studies, many of them drawn from NHANES, participants were asked to recollect the behavior, which named the kind but invited the bias of recollection. Between the two routes the agreement was imperfect, and the disagreement propagated into the outcomes that followed. A further layer was methodological. The compositional and the isotemporal-substitution methods modeled the reallocation of the time and estimated the change in the marker that would accompany it (9, 10); the estimate was a projection from the cross-section rather than an observed effect, a limitation that carried forward into the reading of the results.

#### 3.2.2 Cardiometabolic outcomes

Within community-dwelling older adults, metabolic evidence was predominantly observational, often treating activity, sedentary time, and sleep as a 24-h composition. In the largest study (*n* = 4,562; mean age ≈68 years), greater moderate-to-vigorous activity and sleep were associated with more favorable outcomes across most metabolic markers (all *p* < 0.001), except blood pressure; light activity was associated with waist circumference (B = 0.313, *p* = 0.009) and physical fitness (B = 0.36, *p* < 0.001) (14). Reallocating 30 min of sedentary time to activity or sleep was likewise associated with a better cardiometabolic profile. Smaller compositional studies reported reductions in lean and fat mass with light activity and increases with standing, although triglycerides were the only circulating marker affected (15). Time-use composition was also associated with cardiorespiratory fitness and cardiometabolic risk (16), while movement–sleep profiles distinguished healthy from unhealthy cardiometabolic phenotypes (17). However, these cross-sectional reallocations represent statistical estimates rather than observed intervention effects.

Sedentary time also showed independent associations. Among adults aged ≥65 years who sat for an average of 9.4 h/day, greater sedentary behavior was associated with poorer weight, body-mass index, waist circumference, C-reactive protein, glucose, and functional limitation after adjustment for physical activity (18). Intervention evidence was comparatively limited and inconsistent. Two weeks of step reduction produced only modest metabolic deterioration, with HDL cholesterol declining from 1.62 to 1.55 mmol/L (*p* = 0.017) before returning to baseline after activity resumed (19). Breaking up sitting acutely altered respiratory exchange ratio and non-esterified fatty acids (20), whereas increased free-living activity in older women did not reduce postprandial triglycerides when assessed one day after the final bout (1.78 vs. 1.31 mmol/L, *p* = 0.515) (21).

Activity and sleep were also incorporated into prediction models of resting metabolic rate and body composition (5). Stronger intervention effects emerged from multicomponent aerobic, resistance, and balance training, which improved adropin, nitric oxide, peak oxygen uptake, glucose, insulin, insulin resistance, and blood pressure (22). Walking-related improvements in cardiorespiratory fitness were also linked to more stable sleep in older women (23). Overall, metabolic evidence favored the composition and redistribution of daily behaviors, whereas direct intervention evidence remained limited and heterogeneous.

#### 3.2.3 Well-being outcomes

The well-being literature was larger and more consistent than the metabolic evidence, with cognition as the dominant outcome and sleep frequently acting as both outcome and mediator. Across cross-sectional studies, greater activity and less sedentary time were consistently associated with better cognitive performance. In NHANES, ≥5 h/day of sedentary time predicted lower processing speed (β = −3.1; 95% CI −5.8–−0.4; *p* = 0.02), although the association became non-significant after adjustment for moderate-to-vigorous activity (β = −2.5; 95% CI −5.1–0.2; *p* = 0.07) (24). Similar substitution patterns appeared elsewhere: replacing sedentary time with walking, cycling, or moderate-to-vigorous activity was associated with higher cognitive scores (25), while reallocating sitting to sleep or activity was linked to better self-regulation and executive function (26). Likewise, 24-h time-use composition and contextual behaviors such as recreational activity and television viewing were associated with cognition in the ACTIVate sample (27).

The association was not necessarily linear. In one cross-sectional analysis of short-sleeping older adults drawn from NHANES, cognitive performance improved with exercise volume up to approximately 800–960 MET-min/week before reaching a plateau; below that inflection point, Digit Symbol Substitution Test scores increased by β = 0.555 per 100 MET-min/week (95% CI 0.332–0.778, *p* < 0.001) (28). No other included study tested the same threshold, so the finding should be regarded as hypothesis-generating rather than as a replicated dose ceiling. This dose–response pattern is illustrated conceptually in Figure 3. Activity also commonly formed part of broader lifestyle patterns. The healthiest profile was associated with lower 10-year cognitive-impairment risk (HR = 0.693; 95% CI 0.553–0.868) and approximately 7.5 additional impairment-free years (29). Similar combined effects were reported for physical activity with Mediterranean or Nordic dietary patterns (30, 31), sufficient activity alongside a healthy diet (32), and lifelong activity together with cognitive reserve (33). Overall, physical activity appeared most beneficial as part of a broader behavioral pattern, although this bundling made its independent contribution difficult to isolate.

**Figure 3:**
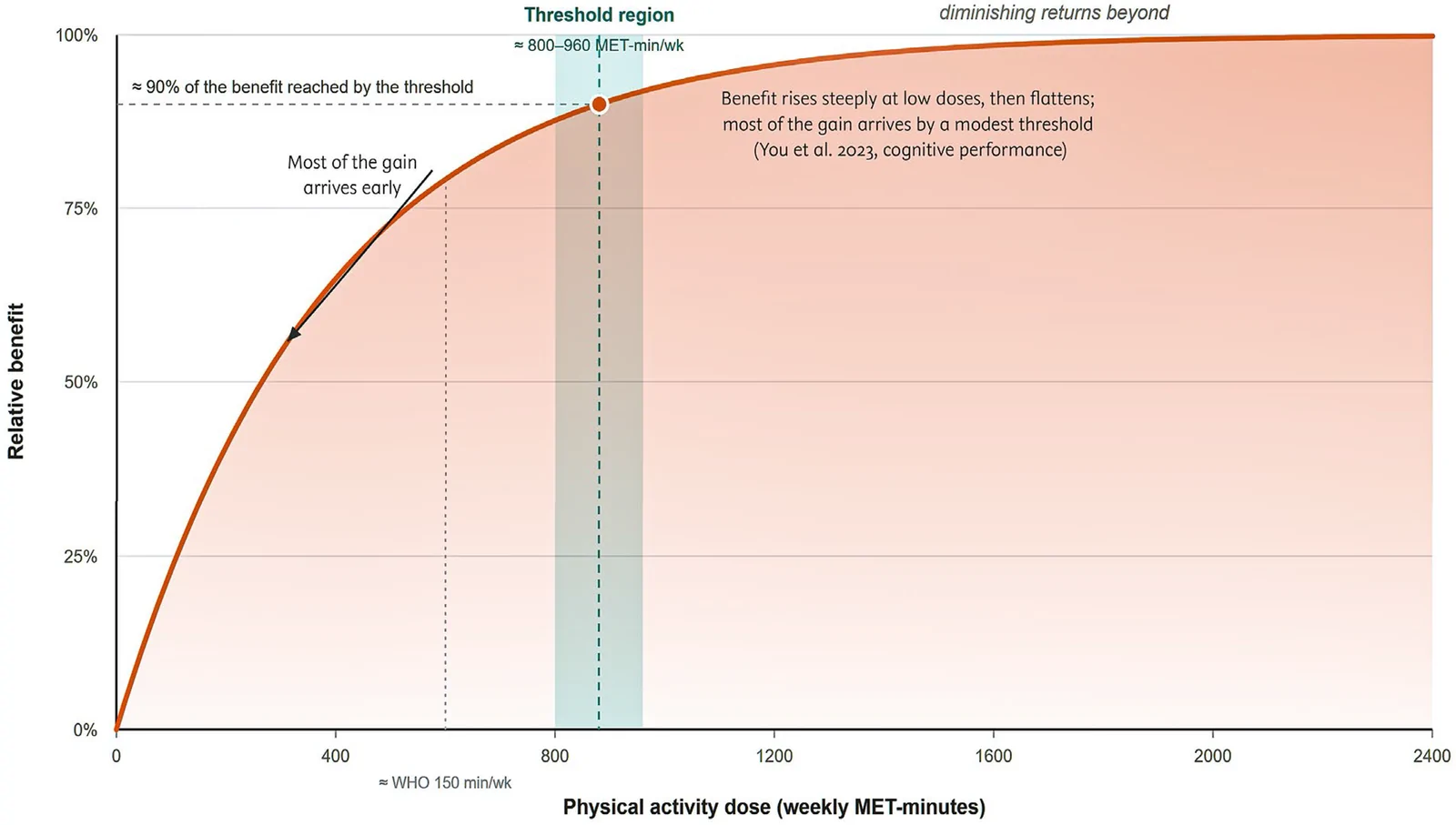
Conceptual dose–response curve for physical activity and cognitive performance in older adults, drawn from a single cross-sectional study. Among short-sleeping older adults in NHANES 2011–2014, cognitive benefit plateaued at approximately 800–960 weekly MET-minutes (MET: metabolic equivalent of task) for both the Digit Symbol Substitution Test and the Animal Fluency Test (28). The curve illustrates the findings of that single study and does not represent a pooled or meta-analytic estimate. A dotted vertical line marks the WHO guideline of 150 min/wk of moderate-intensity activity (7).

Sleep was on both sides of the relationship. In a lifestyle-intervention cohort, exercise and sleep moved together, each one shaping the other across the chronic and the acute time scale (34). Of note, sleep efficiency, and not total sleep time, mediated the link between activity and executive control, working memory, task switching and recall, and age did not moderate that path (35). Sleep carried the effect of a more distant exposure as well: in a community sample, the quality of sleep mediated the path from early-life adversity to later multimorbidity, with lifestyle acting as a moderator (36). Baseline sleep also moderated how much cognition improved with exercise in a further trial (37). On the side of intervention, the signal was cleaner than it had been for metabolism. A walking-dose trial in older women brought the arising time about 9 min earlier on the days after exercise (*p* = 0.01) and steadied the night-to-night sleep (23), and the multicomponent program described above lowered the sleep disorders alongside its metabolic gains (22).

Mood entered through the movement guidelines; older adults who failed to meet the 24-hour movement guidelines carried a higher risk of depression on the PHQ-9 (38). Within four 24-hour activity phenotypes, the proportion of participants with depression symptoms rose from 3.5 to 9.0 percent (*p* = 0.004); the weak and disrupted rhythm carried both the higher odds of depression and the poorer cognition (39). Physical function, the link between the two outcome domains, appeared in the step-reduction trial, where two weeks of reduced walking brought only a limited loss that recovered with rehabilitation (19), and in the sedentary-behavior analysis, where greater sitting tracked with greater functional limitation (18). Quality of life as a named instrument was uncommon in this set.

#### 3.2.4 Assessment and follow-up across studies

The studies differed substantially in how exposures were measured. Several used body-worn devices to quantify movement directly. Accelerometry recorded 9.4 h/day of sedentary time (18), while other studies similarly derived sedentary time, light activity, and sleep from wearable devices (15, 24, 35, 39). Because compositional analyses required complete 24-h time-use data, they also relied on device-based recording (10, 14). Other studies used self-report. Weekly activity, for example, was quantified as questionnaire-derived MET-minutes (28), while lifestyle-profile cohorts constructed exposures from self-reported behaviors (29–31). These approaches were not directly interchangeable, as self-report may overestimate higher-intensity activity and underestimate sedentary time; similarly, actigraphy and sleep diaries can yield different estimates of the same night's sleep.

Outcome measurement was comparatively more consistent. Metabolic studies generally used fasting glucose, insulin, lipids, inflammatory markers, anthropometry, and body-composition assessment (15, 18, 19), while fitness was commonly evaluated through graded exercise testing and peak oxygen uptake (22). Well-being outcomes relied on validated measures, including the Digit Symbol Substitution Test for cognition (28), depression scales for mood (39), and actigraphy or standardized questionnaires for sleep. Quality of life was rarely assessed.

Most studies were cross-sectional, measuring exposure and outcome concurrently and therefore leaving temporal direction unresolved (18, 24, 39). A smaller group used longitudinal designs: one study followed lifestyle profiles and cognitive outcomes over 10 years (29), while another used a similarly extended cohort design (31). Intervention studies covered much shorter periods. Acute laboratory trials assessed metabolic responses within hours (20, 21), one study examined two weeks of step reduction followed by recovery (19), and another evaluated a multicomponent training program (22). In contrast, daily exercise was linked with sleep on the subsequent night (34).

This temporal and methodological heterogeneity limited direct comparison. Acute metabolic shifts, short-term inactivity, and decade-long cognitive change could not be placed on a common scale, while instruments for the same construct often differed across studies. Findings were therefore synthesized narratively rather than pooled and should be interpreted within the measurement method and time window from which each estimate was derived.

### 3.3 Risk of bias of included studies

The risk-of-bias assessment indicated that the methodological quality of the included evidence was heterogeneous (Supplementary Table S3). Of the 27 included studies, three were judged to be at low overall risk of bias, 23 raised some concerns, and one was judged to be at high risk of bias. Among the 18 analytical cross-sectional studies, three were rated as low risk and 15 as having some concerns, with the most frequent limitations relating to self-reported movement or lifestyle exposures, participant selection, and the potential for residual confounding. All three cohort or longitudinal analytical studies raised some concerns, primarily because of self-reported exposures and/or attrition during follow-up. Among the six randomized intervention studies, five raised some concerns, particularly regarding allocation concealment, blinding, adherence, or missing-data handling. One randomized trial was judged to be at high risk because of post-randomization exclusions and analysis of an incomplete randomized sample. Overall, these appraisal findings did not lead to study exclusion but were considered when interpreting the consistency and strength of the mapped evidence.

## 4 Discussion

### 4.1 Summary of main findings

Across the 27 charted studies, the evidence was more substantial and consistent for well-being than for metabolic health. Twenty-two studies reported at least one well-being outcome, 12 included a metabolic marker, and seven addressed both domains. The exposures examined were also unevenly distributed across outcomes. Cognition was assessed across all major exposure categories, including structured exercise, sedentary-time replacement, and multidomain lifestyle patterns, whereas no included study used a named quality-of-life instrument. The direction of evidence similarly favored well-being: cognitive findings were broadly consistent across cross-sectional, longitudinal, and interventional designs, while metabolic responses were more selective, with some markers changing and others remaining unchanged. Compositional and isotemporal approaches were especially prominent in the metabolic literature because they explicitly model the fixed 24-hour behavioral budget (14, 15, 17). Measurement approaches ranged from wearable devices to self-report, and study duration varied from acute laboratory assessments to decade-long follow-up. Two findings were particularly informative across domains: in one cross-sectional study of short-sleeping older adults, the cognitive dose-response appeared to plateau at approximately 800–960 MET-min/week (28), a threshold that awaits replication in other samples; sleep efficiency, rather than sleep duration, emerged as a mediator of the physical activity–cognition relationship in a separate study (35).

### 4.2 Cardiometabolic signal

Among the 12 studies reporting metabolic outcomes, much of the evidence came from compositional analyses treating the day as a 24-hour system. In 4,562 older adults (mean age ≈68 years), a more favorable movement composition was associated with better values for nearly all cardiometabolic markers (all *p* < 0.001), except blood pressure (14). In a smaller cohort, light physical activity and standing were associated with lean and fat mass, while triglycerides were the only circulating marker affected (15). Similarly, 24-hour time-use composition was associated with cardiorespiratory fitness and cardiometabolic risk (16), and movement–sleep profiles distinguished healthier from less healthy cardiometabolic phenotypes (17). Because these studies were cross-sectional, modeled reallocations represent statistical associations rather than observed causal effects.

Sedentary behavior also showed an independent metabolic signal. Among adults aged ≥65 years who accumulated approximately 9.4 h/day of sedentary time, greater sedentary behavior was associated with poorer weight, body mass index, waist circumference, C-reactive protein, and glucose after adjustment for physical activity (18). Intervention findings were less consistent. Two weeks of step reduction lowered HDL cholesterol from 1.62 to 1.55 mmol/L (*p* = 0.017), followed by recovery after rehabilitation (19). Breaking up prolonged sitting acutely altered respiratory exchange ratio (*p* = 0.002) and non-esterified fatty acids (*p* = 0.041) (20), whereas increased free-living activity did not significantly reduce postprandial triglycerides when measured one day after the final bout (1.78 vs. 1.31 mmol/L, *p* = 0.515) (21). These findings suggest that both behavioral exposure and measurement timing influence observed metabolic responses.

Additional evidence remained heterogeneous. Physical activity and sleep were included alongside resting metabolic rate and body composition in a prediction study of adults aged 60–89 years (5). The clearest intervention benefit came from multicomponent aerobic, resistance, and balance training, which increased adropin and nitric oxide while reducing glucose, insulin, insulin resistance, and blood pressure (22). Improved cardiorespiratory fitness was also associated with more stable sleep among older women (23). Overall, evidence was strongest for the daily distribution of movement and sedentary behavior, whereas intervention findings remained limited and mixed.

Studies excluded because they involved clinical or higher-risk populations nevertheless provided contextual support. Sedentary time around meals was associated with postprandial glucose in older adults with overweight (40), while interruptions to prolonged sitting altered cardiometabolic markers and cognition in overweight adults (41). Sleep extension improved metabolic outcomes in obese short sleepers (42), and walking breaks reduced fatigue and altered neuroendocrine responses during prolonged sitting (43). Although outside the review's eligibility criteria, these studies suggest that metabolic effects may be more detectable in populations with greater baseline risk.

### 4.3 Cognition as the dominant well-being outcome

Cognition was the only well-being domain represented across all major exposure categories, whereas quality of life was not assessed with a named instrument, mood appeared in only two studies (38, 39), and physical function in two others (18, 19). Statements about the association between physical activity and “well-being” should therefore be read as applying primarily to cognition; the evidence for mood, physical function, and quality of life was too sparse to support domain-wide conclusions. The cognitive predominance likely reflects both substantive interest and the routine availability of processing-speed tests in large datasets.

Cross-sectional cognitive findings were generally consistent. Sedentary time of ≥5 h/day was associated with a lower Digit Symbol Substitution Test score before adjustment for moderate-to-vigorous physical activity (β = −3.1; 95% CI −5.8–−0.4; *p* = 0.02), but the association became non-significant after adjustment (β = −2.5; 95% CI −5.1–0.2; *p* = 0.07), suggesting that physical activity may partly explain the sedentary behavior–cognition relationship (24). Replacing sedentary time with walking, cycling, or moderate-to-vigorous activity was similarly associated with higher cognitive scores (25), while reallocating sedentary time to sleep or activity was linked to better self-regulation and executive function (26). Twenty-four-hour time use and contextual behaviors, including recreational activity and television viewing, were also associated with cognitive performance (27). In one cross-sectional NHANES analysis restricted to short-sleeping older adults, cognition improved with exercise volume up to approximately 800–960 MET-min/week before plateauing (β = 0.555 per 100 MET-min/week for DSST below the inflection point; 95% CI 0.332–0.778; *p* < 0.001) (28). Because no other included study examined the same dose–response threshold, the finding is best interpreted as a hypothesis for future testing rather than as a confirmed ceiling; it does, however, suggest that the greatest cognitive gain may occur when moving from very low to moderate activity levels.

Physical activity frequently formed part of broader lifestyle patterns. Over 10 years, the healthiest lifestyle profile was associated with lower cognitive-impairment risk (HR = 0.693; 95% CI 0.553–0.868) and approximately 7.5 additional impairment-free years (29). Favorable cognitive outcomes were also associated with a Mediterranean lifestyle (30), an active lifestyle combined with healthy diet (31), sufficient activity alongside healthy dietary behavior (32), and lifelong physical activity together with cognitive reserve (33). Although clinically relevant, these multimodal patterns make the independent contribution of physical activity difficult to isolate.

Evidence from clinical populations outside the eligibility criteria pointed in a similar direction. Among sedentary older adults with obesity, caloric restriction combined with aerobic exercise influenced executive function (44), while greater exercise during weight loss produced larger improvements in reward sensitivity than low-exercise or diet-only approaches (45). An inverted-U association between sleep duration and cognition was also reported in older adults with BMI ≥25 kg/m^2^ (46). Because these three studies enrolled clinical or higher-risk samples, their results were not charted among the 27 included studies; they are cited here solely as contextual support for the direction of the cognitive associations observed in the community-dwelling evidence.

### 4.4 Sleep as mediator, moderator, and outcome

Sleep occupied three distinct positions in the evidence base: mediator, moderator, and outcome. As a mediator, sleep efficiency, rather than total sleep time, mediated the association between physical activity and executive control, working memory, task switching, and recall; age did not moderate this pathway (35). In another domain, sleep quality mediated the relationship between early-life adversity and later multimorbidity, while lifestyle moderated that association (36). Together, these findings emphasize sleep quality and efficiency rather than duration alone.

Sleep also moderated exercise-related outcomes. Baseline sleep quality influenced the magnitude of exercise-induced cognitive change, with greater cognitive gains among participants who slept better at baseline (37). This suggests that sleep status may help explain interindividual variability in cognitive responsiveness to exercise. As an outcome, sleep itself improved in association with activity. Reciprocal day-to-day and longer-term relationships between exercise and subsequent sleep were demonstrated (34). In a walking-dose trial, wake times were approximately 9 min earlier on post-exercise days (*p* = 0.01), and night-to-night sleep variability was reduced with training (23). Fewer sleep disturbances were likewise reported following a multicomponent exercise program alongside metabolic improvement (22).

Evidence from clinical and institutional populations not included in the charted evidence base points in a similar direction. Smart-ring monitoring in older adults with mild cognitive impairment living in a long-term care facility associated light and vigorous activity with less sleep disturbance (47). Exercise training improved sleep quality, sleep efficiency, and depressive symptoms in obese women from a low-resource community (48). Modest sleep restriction did not eliminate the metabolic benefits of exercise in overweight adults (49). Because these studies enrolled participants defined by a clinical condition, institutionalization, or obesity, they were not charted among the 27 included studies; they are introduced here to illustrate that the sleep–activity relationship observed in the community-dwelling evidence extends to higher-risk groups.

### 4.5 Shared biology of metabolism and mind

The findings can be interpreted within an integrated behavioral and biological framework in which physical activity, sedentary behavior, and sleep compete for time within the same 24-hour period and influence both metabolic and neurocognitive outcomes. Physical activity improves insulin sensitivity and glucose regulation, reduces visceral and ectopic adiposity associated with cardiometabolic risk (1), and helps preserve skeletal muscle, whose loss can exacerbate insulin resistance in later life (2). Activity may also influence chronic inflammation, mitochondrial function, and exerkine signaling, pathways with potential relevance to both vascular and brain health.

The included evidence supports sleep as an important component of this shared pathway. Sleep efficiency was identified as a mediator between activity and cognition (35), while baseline sleep quality modified exercise-related cognitive gains (37). From a compositional perspective, the fixed duration of the day means that increasing one behavior necessarily displaces another; thus, replacing sedentary time with light or more intense movement may affect several physiological systems simultaneously. Two studies from outside the charted evidence base offer additional support for that systems view. Using network analysis within a weight-loss intervention in adults with obesity, covariation among physical activity, diet, and sleep-related behaviors was demonstrated (50); similarly, behavioral changes were found to mediate reductions in energy intake among overweight adults (51). Neither study met the community-dwelling eligibility criteria, but both illustrate the interdependence of lifestyle components that the charted findings imply.

### 4.6 Measurement heterogeneity and limits of synthesis

Measurement heterogeneity was a central constraint. Device-based and self-reported physical activity do not represent identical constructs. Accelerometers quantify movement volume and intensity across much of the day but provide limited information about activity context, whereas questionnaires capture modality and context while remaining vulnerable to recall and reporting bias. Device-based estimates were used in several studies (15, 18, 24, 35, 39), whereas weekly activity was quantified in MET-minutes using self-report in another study (28), and multidomain lifestyle cohorts relied substantially on self-reported behaviors (29–31). Because self-report may overestimate higher-intensity activity and underestimate sedentary time, thresholds derived from questionnaires should not be treated as directly equivalent to those derived from accelerometry.

Temporal heterogeneity further limited synthesis. The charted evidence included single-visit cross-sectional studies (18, 24), decade-long cohorts (29, 31), acute laboratory experiments (20, 21), and a two-week step-reduction intervention (19). These designs capture physiological and behavioral processes operating over fundamentally different time scales, making quantitative pooling inappropriate. The present review therefore synthesized findings narratively and comparatively rather than statistically. Emerging technologies may improve future measurement but may also introduce additional methodological variation. Two studies outside the charted evidence base illustrate the possibilities: smart-ring monitoring was used in older adults with mild cognitive impairment living in a long-term care facility (47), while continuous glucose monitoring was combined with sedentary-behavior assessment in older adults with overweight (40). Neither study met the community-dwelling eligibility criteria, but both point toward devices that could strengthen temporal inference if applied in future community-based research. Longer device-based longitudinal studies using standardized protocols could help bridge the current gap between precision measurement and temporal inference

### 4.7 The quality-of-life gap

A notable gap was the absence of named quality-of-life instruments such as the EQ-5D, SF-36, or WHOQOL-BREF across the 27 charted studies. Related evidence in adults aged ≥60 years who exercise regularly indicates that cognitive flexibility and healthy eating attitudes are associated with life satisfaction, underscoring the relevance of patient-reported well-being outcomes in aging research (52). The absence of explicit quality-of-life measurement limits the translational reach of the current evidence because quality of life is a broad outcome with direct relevance to older adults and clinical decision-making.

Two explanations are plausible within the mapped literature. First, investigators may have favored domain-specific outcomes such as cognition, mood, sleep, or physical function rather than generic quality-of-life measures that integrate several dimensions. Second, datasets emphasizing accelerometry, biomarkers, or standardized cognitive testing may provide fewer patient-reported outcomes by design. Future studies should therefore include validated quality-of-life instruments alongside domain-specific endpoints to determine whether observed behavioral and metabolic benefits translate into improvements that are meaningful from the participant perspective.

### 4.8 Strengths and limitations

This scoping review has several strengths. The search covered four databases, the included compositional studies appropriately addressed the fixed 24-hour time budget, and the direction of the cognitive evidence was relatively consistent across heterogeneous designs. At the same time, the limitations are substantial. Much of the charted evidence was cross-sectional, restricting causal interpretation (18, 24). Physical activity and sedentary behavior were assessed using both wearable devices and self-report, which measure different aspects of exposure and hinder direct comparison or pooling. Quality of life was not directly represented among the charted outcomes, as discussed in the Quality-of-Life Gap section. No review protocol was prospectively registered before data extraction commenced, which means that deviations from the original plan, if any occurred, cannot be verified against a public record; future scoping reviews in this area would benefit from prospective registration on PROSPERO or the Open Science Framework. In addition, the risk-of-bias profile of the charted evidence was modest overall: only three of the 27 included studies were rated at low risk, 23 raised some concerns, and one was judged at high risk. The predominance of the “some concerns” category, driven largely by self-reported exposures and incomplete confounder control, means that the consistency of the cognitive findings should be interpreted alongside this methodological heterogeneity rather than taken as strong confirmatory evidence.

The metabolic evidence was also comparatively sparse and depended heavily on observational compositional studies, with only limited intervention evidence. Multidomain lifestyle studies further complicate attribution because physical activity was embedded within dietary, cognitive, social, or other behavioral components (29–31). Finally, restricting eligibility to community-dwelling populations excluded 16 studies involving clinical or higher-risk samples that were discussed contextually and in which metabolic effects sometimes appeared more pronounced. This restriction was consistent with the review scope but limits generalizability to older adults with substantial metabolic disease, obesity, institutionalization, or other clinical characteristics.

### 4.9 Future research directions

Future research should move beyond predominantly cross-sectional evidence toward studies designed to test the 24-hour compositional question directly. Reallocation trials that experimentally shift time between sedentary behavior, light activity, moderate-to-vigorous activity, and sleep, while using device-based exposure measurement and sufficiently long follow-up, would provide stronger evidence regarding metabolic consequences. Studies adequately powered for glycaemia, lipid outcomes, and incident dysglycaemia are particularly needed in older adults. Quality of life and other patient-reported outcomes should be incorporated routinely so that physiological and cognitive changes can be linked to outcomes that matter directly to participants.

Factorial or otherwise component-specific designs could help separate the contribution of physical activity from diet, social engagement, and cognitive stimulation within multidomain lifestyle interventions. Device-based longitudinal cohorts would also strengthen temporal inference. Wearable technologies, smart rings, and continuous glucose monitoring have begun to appear in pilot work (40, 47) and may permit more precise characterization of 24-hour behavior if applied over longer periods with standardized methods. Mechanistic studies integrating exerkines and metabolomic profiling with clinical outcomes could further clarify the biological pathways linking movement to metabolic and neurocognitive health (5, 8). Finally, future research should intentionally include under-represented groups, particularly the oldest-old and multimorbid populations, and should consider stratified analyses that place community-dwelling and higher-risk clinical populations side by side

## 5 Conclusions

This scoping review indicates that physical activity, sedentary behavior, and related 24-hour movement patterns are associated with both cardiometabolic health and well-being in community-dwelling older adults, although the strength of evidence varies across these domains. Evidence relating to well-being was broader and more consistent, although it was dominated by cognition; mood, physical function, and quality of life were assessed too infrequently to support conclusions about well-being as a whole. Metabolic findings were less consistent and relied largely on compositional and cross-sectional analyses, while intervention evidence remained relatively limited.

The available evidence points to greater physical activity, less prolonged sedentary behavior, and adequate, efficient sleep as closely related features of healthy aging. These associations, however, should not be interpreted as evidence of uniform causal effects across metabolic and well-being outcomes. Much of the literature remains limited by cross-sectional designs, differences in exposure measurement, scarce assessment of quality of life, and a shortage of long-term intervention studies. Future research would benefit from more consistent device-based measurement, direct experimental testing of time reallocation between daily behaviors, greater use of patient-reported outcomes, and longer follow-up periods. Such work may clarify which changes in daily movement patterns produce clinically meaningful benefits for metabolic health and well-being in older adults.

## Data Availability

The original contributions presented in the study are included in the article/Supplementary Material, further inquiries can be directed to the corresponding author.
