# Supplementary material for: Physical activity, metabolic health and well-being in community-dwelling older adults: a scoping review
Source: Front Med (Lausanne). 2026 Oct 16;13:1969460. doi: 10.3389/fmed.2026.1969460 (PMC13719392; doi:10.3389/fmed.2026.1969460)
Supplement: Supplementary file 1 [file Table1.docx]

***Supplementary Material***

**Table S1. Full search strategy for each electronic database.**

Database-specific searches combined population terms referring to older and community-dwelling adults with movement-behavior and health-related terms; where applicable, study-design terms and database-specific filters were also used. The exact database-specific queries are reproduced below as executed, together with field codes, limits, filters, and result counts. All searches were conducted in May 2026.

**PubMed**

*Field: [tiab] (title and abstract). Filters: english[lang], 2010:2026[pdat], humans[mesh].*

| **Pass** | **Search query** | **Results** |
| --- | --- | --- |
| **A** | (“sleep”[tiab] OR “sleep duration”[tiab] OR “sleep quality”[tiab]) OR (“older adults”[tiab] OR “older people”[tiab] OR elderly[tiab] OR aged[tiab] OR “community-dwelling”[tiab] OR geriatric[tiab] OR “older men”[tiab] OR “older women”[tiab] OR seniors[tiab]) AND (“physical activity”[tiab] OR exercise[tiab] OR “resistance training”[tiab] OR “aerobic training”[tiab] OR “endurance training”[tiab] OR “physical fitness”[tiab] OR “sedentary behavior”[tiab] OR “sedentary behaviour”[tiab] OR “muscle strengthening”[tiab] OR “exercise training”[tiab]) AND (“metabolic health”[tiab] OR “metabolic syndrome”[tiab] OR “insulin sensitivity”[tiab] OR “insulin resistance”[tiab] OR “body composition”[tiab] OR glyc*[tiab] OR glucose[tiab] OR HbA1c[tiab] OR “glycated hemoglobin”[tiab] OR lipid*[tiab] OR triglyceride*[tiab] OR cholesterol[tiab] OR cardiometabolic[tiab] OR sarcopenia[tiab] OR adipokine*[tiab] OR inflammation[tiab] OR “C-reactive protein”[tiab]) AND (“randomized controlled trial”[tiab] OR “controlled trial”[tiab] OR “clinical trial”[tiab] OR “meta-analysis”[tiab] OR “systematic review”[tiab] OR “network meta-analysis”[tiab] OR cohort[tiab] OR “observational study”[tiab] OR “longitudinal study”[tiab] OR “cross-sectional”[tiab]) AND (english[lang]) AND (2010:2026[pdat]) AND (humans[mesh]) | 1908 |
| **B** | (“older adults”[tiab] OR “older people”[tiab] OR elderly[tiab] OR aged[tiab] OR “community-dwelling”[tiab] OR geriatric[tiab] OR “older men”[tiab] OR “older women”[tiab] OR seniors[tiab]) AND (“physical activity”[tiab] OR exercise[tiab] OR “resistance training”[tiab] OR “aerobic training”[tiab] OR “endurance training”[tiab] OR “physical fitness”[tiab] OR “sedentary behavior”[tiab] OR “sedentary behaviour”[tiab] OR “muscle strengthening”[tiab] OR “exercise training”[tiab]) AND (“metabolic health”[tiab] OR “metabolic syndrome”[tiab] OR “insulin sensitivity”[tiab] OR “insulin resistance”[tiab] OR “body composition”[tiab] OR glyc*[tiab] OR glucose[tiab] OR HbA1c[tiab] OR “glycated hemoglobin”[tiab] OR lipid*[tiab] OR triglyceride*[tiab] OR cholesterol[tiab] OR cardiometabolic[tiab] OR sarcopenia[tiab] OR adipokine*[tiab] OR inflammation[tiab] OR “C-reactive protein”[tiab]) AND (english[lang]) AND (2010:2026[pdat]) AND (humans[mesh]) | 1128 |
| **Database subtotal** | | **3036** |

**Web of Science (Core Collection)**

*Refined by: Publication Years 2010–2026, Languages English, Document Types Article or Review.*

| **Pass** | **Search query** | **Results** |
| --- | --- | --- |
| **A** | TS=(“older adult*” OR “older people” OR elderly OR “community-dwelling” OR geriatric OR “older men” OR “older women” OR senior*) AND TS=(“physical activity” OR exercise OR “resistance training” OR “aerobic training” OR “endurance training” OR “physical fitness” OR “sedentary behavio*” OR “muscle strengthening” OR “exercise training”) AND TS=(“metabolic health” OR “metabolic syndrome” OR “insulin sensitivity” OR “insulin resistance” OR “body composition” OR glyc* OR glucose OR HbA1c OR “glycated h?emoglobin” OR lipid* OR triglyceride* OR cholesterol OR cardiometabolic OR sarcopenia OR adipokine* OR inflammation OR “C-reactive protein”) AND TS=(“randomized controlled trial” OR “controlled trial” OR “clinical trial” OR “meta-analysis” OR “systematic review” OR “network meta-analysis” OR cohort OR “observational study” OR “longitudinal study” OR “cross-sectional”) | 2435 |
| **B** | TS=(“older adult*” OR “older people” OR elderly OR “community-dwelling” OR geriatric OR “older men” OR “older women” OR senior*) AND TS=(“physical activity” OR exercise OR “resistance training” OR “aerobic training” OR “endurance training” OR “physical fitness” OR “sedentary behavio*” OR “muscle strengthening” OR “exercise training”) AND TS=(“metabolic health” OR “metabolic syndrome” OR “insulin sensitivity” OR “insulin resistance” OR “body composition” OR glyc* OR glucose OR HbA1c OR “glycated h?emoglobin” OR lipid* OR triglyceride* OR cholesterol OR cardiometabolic OR sarcopenia OR adipokine* OR inflammation OR “C-reactive protein”) AND NOT TS=(“randomized controlled trial” OR “controlled trial” OR “clinical trial” OR “meta-analysis” OR “systematic review” OR “network meta-analysis” OR cohort OR “observational study” OR “longitudinal study” OR “cross-sectional”) | 3630 |
| **Database subtotal** | | **6065** |

**Scopus**

*Field: TITLE-ABS (title and abstract only). Filters: PUBYEAR > 2009 AND PUBYEAR < 2027, English, Article or Review, preprints excluded.*

| **Pass** | **Search query** | **Results** |
| --- | --- | --- |
| **A** | TITLE-ABS(“older adult*” OR “older people” OR elderly OR aged OR “community-dwelling” OR geriatric OR “older men” OR “older women” OR senior*) AND TITLE-ABS(“physical activity” OR exercise OR “resistance training” OR “aerobic training” OR “endurance training” OR “physical fitness” OR “sedentary behavio*” OR “muscle strengthening” OR “exercise training”) AND TITLE-ABS(“metabolic health” OR “metabolic syndrome” OR “insulin sensitivity” OR “insulin resistance” OR “body composition” OR glyc* OR glucose OR HbA1c OR “glycated hemoglobin” OR “glycated haemoglobin” OR lipid* OR triglyceride* OR cholesterol OR cardiometabolic OR sarcopenia OR adipokine* OR inflammation OR “C-reactive protein”) AND TITLE-ABS(“randomized controlled trial” OR “controlled trial” OR “clinical trial” OR “meta-analysis” OR “systematic review” OR “network meta-analysis” OR cohort OR “observational study” OR “longitudinal study” OR “cross-sectional”) AND PUBYEAR > 2009 AND PUBYEAR < 2027 AND (LANGUAGE(english)) AND (DOCTYPE(ar) OR DOCTYPE(re)) | 5802 |
| **B** | TITLE-ABS(“older adult*” OR “older people” OR elderly OR aged OR “community-dwelling” OR geriatric OR “older men” OR “older women” OR senior*) AND TITLE-ABS(“physical activity” OR exercise OR “resistance training” OR “aerobic training” OR “endurance training” OR “physical fitness” OR “sedentary behavio*” OR “muscle strengthening” OR “exercise training”) AND TITLE-ABS(“metabolic health” OR “metabolic syndrome” OR “insulin sensitivity” OR “insulin resistance” OR “body composition” OR glyc* OR glucose OR HbA1c OR “glycated hemoglobin” OR “glycated haemoglobin” OR lipid* OR triglyceride* OR cholesterol OR cardiometabolic OR sarcopenia OR adipokine* OR inflammation OR “C-reactive protein”) AND NOT TITLE-ABS(“randomized controlled trial” OR “controlled trial” OR “clinical trial” OR “meta-analysis” OR “systematic review” OR “network meta-analysis” OR cohort OR “observational study” OR “longitudinal study” OR “cross-sectional”) AND PUBYEAR > 2009 AND PUBYEAR < 2027 AND (LANGUAGE(english)) AND (DOCTYPE(ar) OR DOCTYPE(re)) | 5238 |
| **Database subtotal** | | **11040** |

**SPORTDiscus (via EBSCO)**

*Limiters: Academic Journals, Article, English, 2010–2026, Peer Reviewed. Search mode: title or abstract.*

| **Pass** | **Search query** | **Results** |
| --- | --- | --- |
| **A** | (“older adults” OR “older people” OR elderly OR aged OR “community-dwelling” OR geriatric OR “older men” OR “older women” OR seniors) AND (“physical activity” OR exercise OR “resistance training” OR “aerobic training” OR “endurance training” OR “physical fitness” OR “sedentary behavior” OR “sedentary behaviour” OR “muscle strengthening” OR “exercise training”) AND (“metabolic health” OR “metabolic syndrome” OR “insulin sensitivity” OR “insulin resistance” OR “body composition” OR glyc* OR glucose OR HbA1c OR “glycated hemoglobin” OR lipid* OR triglyceride* OR cholesterol OR cardiometabolic OR sarcopenia OR adipokine* OR inflammation OR “C-reactive protein”) AND (“randomized controlled trial” OR “controlled trial” OR “clinical trial” OR “meta-analysis” OR “systematic review” OR “network meta-analysis” OR cohort OR “observational study” OR “longitudinal study” OR “cross-sectional”) | 672 |
| **B** | (“older adults” OR “older people” OR elderly OR aged OR “community-dwelling” OR geriatric OR “older men” OR “older women” OR seniors) AND (“physical activity” OR exercise OR “resistance training” OR “aerobic training” OR “endurance training” OR “physical fitness” OR “sedentary behavior” OR “sedentary behaviour” OR “muscle strengthening” OR “exercise training”) AND (“metabolic health” OR “metabolic syndrome” OR “insulin sensitivity” OR “insulin resistance” OR “body composition” OR glyc* OR glucose OR HbA1c OR “glycated hemoglobin” OR lipid* OR triglyceride* OR cholesterol OR cardiometabolic OR sarcopenia OR adipokine* OR inflammation OR “C-reactive protein”) | 1182 |
| **Database subtotal** | | **1854** |

**Combined identification total: 21,995 records across four databases before de-duplication.**

Reference-list hand-searching yielded 193 citations for supplementary screening. Of these, 190 did not proceed to full-text retrieval following citation-level screening, and three potentially eligible reports were retrieved for full-text assessment. The citation-searching pathway is presented in the PRISMA flow diagram under “Identification of studies via other methods.”
