# Supplementary material for: Physical activity, metabolic health and well-being in community-dwelling older adults: a scoping review
Source: Front Med (Lausanne). 2026 Oct 16;13:1969460. doi: 10.3389/fmed.2026.1969460 (PMC13719392; doi:10.3389/fmed.2026.1969460)
Supplement: Supplementary file 2 [file Table2.docx]

Supplementary Material

**Table S2. Full-text reports not included in the charted evidence base (n = 55).**

Of the 82 full-text reports assessed for eligibility, 55 did not enter the charted evidence base. Among these, 39 did not meet the charting criteria. A further 16 were not counted among the 27 included studies but were retained solely as clinical or contextual references for the Discussion because their sampling frames, intervention contexts, or study populations lay outside the core charted evidence set.

**A. Reports excluded on charting criteria (n = 39)**

| **Study** | **Title** | **Reason for non-inclusion** |
| --- | --- | --- |
| Al Khatib, HK (2018) | *Sleep extension is a feasible lifestyle intervention in free-living adults who are habitually short sleepers: a potential strategy for decreasing intake of free sugars? A randomized controlled pilot study* | Adults 18-64; outside older-adult scope. |
| Baron, KG (2023) | *Circadian Timing, Information processing and Metabolism (TIME) study: protocol of a longitudinal study of sleep duration, circadian alignment and cardiometabolic health among overweight adults* | Protocol only; overweight adults; no completed outcomes. |
| Basso, JC (2022) | *Examining the Effect of Increased Aerobic Exercise in Moderately Fit Adults on Psychological State and Cognitive Function* | Moderately fit adults aged 25-59; outside older-adult scope. |
| Beathard, KM (2023) | *The impact of nutrition on visual cognitive performance in the nutrition, vision, and cognition in sport study* | Young healthy adults/sport-vision nutrition study; outside older-adult scope. |
| Bender, DV (2012) | *Nutritional and Behavioral Modification Therapies of Obesity: Facts and Fiction* | Review/clinical guidance on obesity, not a primary older-adult study. |
| Buman, MP (2014) | *Reallocating Time to Sleep, Sedentary Behaviors, or Active Behaviors: Associations With Cardiovascular Disease Risk Biomarkers, NHANES 2005-2006* | NHANES adult sample; not older-adult specific. |
| Campos-Hernandez C (2026) | *Effects of 12-Week Multicomponent Training Program on Body Composition, Metabolic Health, and Physical Performance in Middle-Aged and Older Women: Exploratory Role of Baseline Adiposity* | Age range 50-72 and mixed middle-aged/older sample; exclude from older-adult chart unless full text provides older-only subgroup. |
| Castelli, D (2023) | *Physical Activity Interventions to Reduce Metabolic Risk Factors to Cognitive Health* | Transdisciplinary/school-based intervention development paper; not older adults and not chartable primary evidence. |
| Charlett, OP (2021) | *Impaired postprandial glucose and no improvement in other cardiometabolic responses or cognitive function by breaking up sitting with bodyweight resistance exercises: a randomised crossover trial* | Young healthy adults (25 ± 6 y); outside older-adult scope. |
| Demetriou, CA (2025) | *Associations Between Chrono-Nutrition Behaviours and Cognitive Function in Middle-Aged Adults: The NUTRICO Cross-Sectional Cohort Study* | Adults 45-65, median 52; outside older-adult scope. |
| Deru, LS (2025) | *The effects of alternate-day fasting on sleep and physical activity in poor sleeping adults: A randomized control trial* | Healthy young adults (mean 22.5 y); outside older-adult scope. |
| Dunstan, DW (2018) | *Interacting effects of exercise with breaks in sitting time on cognitive and metabolic function in older adults: Rationale and design of a randomised crossover trial* | Protocol/rationale paper; no completed trial outcomes. |
| Evenson, KR (2014) | *Physical Activity and Sedentary Behavior Among Adults 60 Years and Older: New York City Residents Compared With a National Sample* | Descriptive PA/sedentary prevalence comparison only; useful for background, not outcome chart. |
| Farrell SW (2021) | *Functional Movement Screening Performance and Association With Key Health Markers in Older Adults* | Functional Movement Screen performance was the primary exposure; physical activity was examined as a correlate/health marker rather than as the movement-behavior exposure of interest. Therefore, the study did not meet the exposure–outcome eligibility criteria for the charted evidence base. |
| Gijselaers, HJM (2016) | *Physical Activity, Sleep, and Nutrition Do Not Predict Cognitive Performance in Young and Middle -Aged Adults* | Young and middle-aged adults; outside older-adult scope. |
| Gooderham, GK (2025) | *Metacognitive function in young adults is impacted by physical activity, diet, and sleep patterns* | Young-adult metacognition study; outside older-adult scope. |
| Guo, PY (2026) | *Adiposity, diet or inactivity - which is the culprit in obesity-related memory deficits in human adults?* | Adults 19-47; outside older-adult scope. |
| Hooker, SA (2020) | *Do Physical Activity, Caloric Intake, and Sleep Vary Together Day to Day? Exploration of Intraindividual Variability in 3 Key Health Behaviors* | Healthy adult daily-behavior variability study; older-adult sampling frame not stated. |
| Iacovides, S (2019) | *Three consecutive weeks of nutritional ketosis has no effect on cognitive function, sleep, and mood compared with a high-carbohydrate, low-fat diet in healthy individuals: a randomized, crossover, controlled trial* | Healthy normal-weight adults, mean 30 y; outside older-adult scope. |
| Keadle, SK (2017) | *Targeting Reductions in Sitting Time to Increase Physical Activity and Improve Health* | Knowledge update/non-primary review; use as background only if needed. |
| Lewis, ED (2023) | *A Multi-Center, Open-Label Exploratory Study to Assess Cognitive Function Response to Lifestyle Changes Plus Supplementation in Healthy Adults with Risk Factors Associated with Cognitive Decline* | Adults ≥45 at cognitive-risk; not older-adult community sample and open-label supplement/lifestyle protocol. |
| Malaikah, S (2025) | *Replacing Sedentary Time with Physical Activity and Sleep: A 24-Hour Movement Behaviour Perspective on Appetite Control* | Young healthy active adults; outside older-adult scope. |
| Marlatt, KL (2017) | *Persistence of weight loss and acquired behaviors 2 y after stopping a 2-y calorie restriction intervention* | CALERIE nonobese adult calorie-restriction follow-up; not older-adult specific. |
| Mendelson, M (2026) | *Study protocol for a theory-informed randomized controlled trial of a lifestyle and sleep intervention to improve quality of life and physical activity in inactive adults: the SleeP exercIse nutRition heALth+ SPIRAL+ study* | Protocol only and adults 18-80; no completed outcome data. |
| Miao, M (2017) | *Carry-over effect between diet and physical activity: the bottom-up and top-down hypotheses of hierarchical self-efficacy* | General adult self-efficacy behavior-change study; outside older-adult scope. |
| Miladi, S (2026) | *The combination of physical activity and late-time-restricted eating improves eating behavior, cognitive performance and cardiovascular parameters in women living with excess body weight* | Women with overweight/obesity, mean ages in 20s-30s; outside older-adult scope. |
| Mues, JP (2025) | *The Influence of Workplace-Integrated Exercise Snacks on Cognitive Performance in Sedentary Middle-Aged Adults-A Randomized Pilot Study* | Sedentary middle-aged office workers; outside older-adult scope. |
| Parker, KRB (2025) | *Increases in adherence to a simplified Mediterranean diet predicts improved next day cognitive functioning* | Middle-aged and older adults, mean age 54; outside older-adult chart scope. |
| Ptomey, LT (2022) | *The impact of exercise and cumulative physical activity on energy intake and diet quality in adults enrolled in the Midwest Exercise Trial for the Prevention of Weight Regain* | Adults mean age 42.5 y in exercise/energy-intake trial; outside older-adult scope. |
| Quinlan, C (2021) | *The accuracy of self-reported physical activity questionnaires varies with sex and body mass index* | Middle-aged measurement-validity study; outside older-adult scope. |
| Ratz, T (2021) | *Health-Related Lifestyle and Dropout From a Web-Based Physical Activity Intervention Trial in Older Adults: A Latent Profile Analysis* | Dropout/adherence from web-based PA intervention; no metabolic or well-being outcome for charting. |
| Roca-Ventura, A (2025) | *Clustering on longitudinal lifestyle trajectories and their impact on cognitive performance* | Healthy middle-aged cohort; outside older-adult scope. |
| Saleh, D (2014) | *Interrelationships among sedentary time, sleep duration, and the metabolic syndrome in adults* | NHANES adults aged ≥20; not older-adult specific. |
| Serra, MC (2020) | *Healthy Lifestyle and Cognition: Interaction between Diet and Physical Activity* | Narrative review/non-primary article; do not chart as empirical evidence. |
| Stamatakis, E (2025) | *Minimum and optimal combined variations in sleep, physical activity, and nutrition in relation to all-cause mortality risk* | Large UK Biobank SPAN-mortality analysis; outcome is mortality, not metabolic or well-being; peripheral background only. |
| Wiedemann, RG (2014) | *Self-reported physical activity is associated with cognitive function in lean, but not obese individuals* | College students; outside older-adult scope. |
| You, YW (2023) | *The association between recreational physical activity and depression in the short sleep population: a cross-sectional study* | Short-sleep adult NHANES sample; not older-adult specific. |
| You, YW (2024) | *Inverted U-shaped relationship between sleep duration and phenotypic age in US adults: a population-based study* | US adult phenotypic-age analysis; not older-adult specific. |
| Zeng, JF (2025) | *The association between weekend catch-up sleep and obesity in US adults: A cross-sectional analysis of NHANES 2017-2020* | Adult obesity/sleep analysis without PA focus; outside chart scope. |

**B. Clinical or contextual reports retained for discussion only (n = 16)**

| **Study** | **Title** | **Reason for non-inclusion** |
| --- | --- | --- |
| Chevance, G (2020) | *Modelling multiple health behavior change with network analyses: results from a one-year study conducted among overweight and obese adults* | Clinical obesity/behavior-change frame; retained for contextual discussion only. |
| Chun, E (2024) | *Pre- Versus Postmeal Sedentary Duration-Impact on Postprandial Glucose in Older Adults With Overweight or Obesity* | Higher-risk overweight/obesity sampling frame; retained for contextual discussion only. |
| Creasy, SA (2025) | *Effect of physical activity on changes in weight and aerobic capacity during an 18-month behavioral weight loss intervention* | Behavioral weight-loss/obesity intervention frame; retained for contextual discussion only. |
| Crochiere, RJ (2026) | *Moderators of the Relation Between Physical Activity and Subsequent Energy Intake Among Behavioral Weight Loss Participants* | Behavioral weight-loss intervention frame; retained for contextual discussion only. |
| Errickson, SP (2016) | *Improvements in Functional Exercise Capacity after a Residential Behavioural Change, Diet and Fitness Program for Obese Adults* | Residential obesity-treatment program; retained for contextual discussion only. |
| Fenton, S (2021) | *Behavioural mediators of reduced energy intake in a physical activity, diet, and sleep behaviour weight loss intervention in adults* | Behavioral weight-loss intervention frame; retained for contextual discussion only. |
| Hartescu, I (2022) | *Sleep extension and metabolic health in male overweight/obese short sleepers: A randomised controlled trial* | Overweight/obesity short-sleeper clinical sample; retained for contextual discussion only. |
| Hugenschmidt, CE (2019) | *Cognitive Effects of Adding Caloric Restriction to Aerobic Exercise Training in Older Adults with Obesity* | Older adults with obesity in a caloric-restriction/exercise intervention; retained for contextual discussion only. |
| Lee, J (2026) | *Smart ring-based assessment of physical activity intensity and sleep disturbances in older adults with mild cognitive impairment* | Long-term-care and MCI-defined sample; not community-dwelling; retained for contextual discussion only. |
| Mendham, AE (2021) | *Improved Sleep Quality and Depressive Symptoms With Exercise Training in Obese Women From a Low Socioeconomic Community: A Randomized Controlled Trial* | Clinical obesity exercise sample; retained for contextual discussion only. |
| Peven, JC (2020) | *The Effects of a 12-Month Weight Loss Intervention on Cognitive Outcomes in Adults with Overweight and Obesity* | Behavioral weight-loss intervention in adults with overweight/obesity; retained for contextual discussion only. |
| Porter, JW (2021) | *Modest sleep restriction does not influence steps, physical activity intensity or glucose tolerance in obese adults* | Adult obesity sample; retained for contextual discussion only. |
| Qiu, KY (2025) | *Threshold effects of sleep duration and cognitive function in older adults with BMI ≥ 25 kg/m2* | Higher-risk BMI-defined older-adult subgroup; retained for contextual discussion only. |
| Wanders, L (2021) | *Impact of prolonged sitting and physical activity breaks on cognitive performance, perceivable benefits, and cardiometabolic health in overweight/obese adults: The role of meal composition* | Overweight/obesity experimental sample; retained for contextual discussion only. |
| Wang, XW (2018) | *Influence of sleep restriction on weight loss outcomes associated with caloric restriction* | Caloric-restriction/weight-loss intervention context; retained for contextual discussion only. |
| Wennberg, P (2016) | *Acute effects of breaking up prolonged sitting on fatigue and cognition: a pilot study* | Overweight/obesity experimental sample; retained for contextual discussion only. |
