# Supplementary material for: Physical activity, metabolic health and well-being in community-dwelling older adults: a scoping review
Source: Front Med (Lausanne). 2026 Oct 16;13:1969460. doi: 10.3389/fmed.2026.1969460 (PMC13719392; doi:10.3389/fmed.2026.1969460)
Supplement: Supplementary file 3 [file Table3.docx]

Supplementary Material

**Table S3. Study-level risk-of-bias assessment of the included studies (n = 27).**

Risk of bias was appraised according to the analytic design of each included report. Analytical cross-sectional and cohort/longitudinal studies were appraised using the corresponding revised JBI tools; parallel-group randomized trials were appraised using the revised JBI randomized controlled trial tool; the randomized crossover trial was appraised using the Cochrane RoB 2 crossover framework. Judgments are presented as Low risk, Some concerns, High risk, or Unclear. No study was excluded on the basis of this appraisal. Overall, 3 studies were judged to be at low risk, 23 raised some concerns, and 1 was judged to be at high risk.

A. Analytical cross-sectional studies (n = 18)

| **Study** | **Selection** | **Exposure** | **Confounding** | **Outcome** | **Analysis** | **Overall** | **Main appraisal considerations** |
| --- | --- | --- | --- | --- | --- | --- | --- |
| Anastasiou et al. (2018) | Low | Some concerns | Low | Low | Low | Some concerns | Population-based random sampling and comprehensive cognitive assessment; lifestyle exposure was based on validated questionnaires, leaving potential self-report measurement error. |
| Dong et al. (2026) | Some concerns | Some concerns | Some concerns | Low | Low | Some concerns | Community-based cross-sectional prediction study; physical activity and sleep were questionnaire-derived predictors/covariates rather than the principal exposure, with potential residual confounding. |
| Dumuid et al. (2018b) | Some concerns | Low | Low | Low | Low | Some concerns | Appropriate compositional/isotemporal analysis of 24-hour behaviors and cardiometabolic outcomes; participant selection and the cross-sectional sampling frame limit representativeness and causal interpretation. |
| Edwards and Loprinzi (2017) | Low | Some concerns | Low | Low | Low | Some concerns | Large NHANES sample and standardized cognition outcome; sedentary behavior and physical activity were self-reported, increasing exposure misclassification risk. |
| Fanning et al. (2017) | Some concerns | Some concerns | Low | Low | Low | Some concerns | Baseline analysis of low-active volunteers recruited to an exercise trial; accelerometry strengthened activity measurement, but sleep was self-reported and outcome data were incomplete for some cognitive tasks. |
| Gennuso et al. (2013) | Low | Low | Low | Low | Low | Low risk | Large population-based NHANES sample, device-measured sedentary behavior/physical activity, standardized biomarkers and functional measures, and multivariable adjustment. |
| Liang et al. (2024) | Low | Low | Low | Low | Low | Low risk | Large population-based sample with 24-hour compositional modeling, multiple health outcomes, covariate adjustment, and sensitivity analyses; cross-sectional temporality remains a design limitation rather than a major internal-bias signal. |
| Mellow et al. (2022) | Some concerns | Low | Low | Low | Low | Some concerns | Seven-day triaxial accelerometry and validated cognitive battery with covariate and false-discovery-rate adjustment; volunteer baseline cohort and some contextual behaviors/sleep quality relied on self-report. |
| Nijholt et al. (2016) | Some concerns | Some concerns | Low | Low | Low | Some concerns | Large older-adult sample with adjusted analyses and standardized cognition; physical activity and diet were self-reported, creating exposure measurement concerns. |
| Powell et al. (2020) | Some concerns | Low | Low | Low | Low | Some concerns | Device-based 24-hour behavior assessment and objective cardiometabolic measures with compositional analysis; cohort recruitment/selection and residual confounding remain potential concerns. |
| Ryan et al. (2019) | Some concerns | Some concerns | Some concerns | Low | Low | Some concerns | Objective free-living activity measurement and fasting biomarkers; small community sample (n = 93), self-reported sleep, and limited precision/possible residual confounding. |
| Scotti et al. (2026) | Some concerns | Some concerns | Low | Low | Low | Some concerns | Standardized remote neuropsychological testing and covariate-adjusted models; convenience/network recruitment and self-reported lifestyle exposures may introduce selection and measurement bias. |
| Sheffler et al. (2025) | Some concerns | Some concerns | Low | Some concerns | Low | Some concerns | Multivariable mediation/moderation modeling across community samples; key lifestyle/sleep and health constructs were largely self-reported and the cross-sectional component limits directionality. |
| Smagula et al. (2022) | Low | Low | Low | Low | Low | Low risk | Representative NHANES sampling, continuous wrist accelerometry, validated PHQ-9/cognitive measures, sample weighting and prespecified covariate-adjusted analyses. |
| Wei et al. (2021) | Low | Some concerns | Low | Low | Low | Some concerns | Large NHANES sample, standardized cognitive tests and isotemporal multivariable models; sleep and activity behaviors were interview/self-report measures. |
| Wilckens et al. (2018) | Some concerns | Low | Low | Low | Low | Some concerns | One-week accelerometry for activity and sleep and laboratory cognition with covariate-adjusted bootstrap mediation; modest older-adult subgroup and cross-sectional mediation constrain causal interpretation. |
| You et al. (2023) | Low | Some concerns | Low | Low | Low | Some concerns | NHANES probability sample with weighted multivariable and threshold analyses; exercise/sleep exposure information was self-reported and complete-case selection may introduce bias. |
| Zamora et al. (2025) | Low | Some concerns | Low | Low | Low | Some concerns | Population-based NHANES analysis with validated depression outcome and adjusted regression; movement-guideline adherence relied mainly on self-reported sleep, sedentary behavior and activity. |

B. Cohort and longitudinal analytical studies (n = 3)

| **Study** | **Selection** | **Exposure** | **Confounding** | **Outcome** | **Follow-up / missing data** | **Analysis** | **Overall** | **Main appraisal considerations** |
| --- | --- | --- | --- | --- | --- | --- | --- | --- |
| Hu et al. (2025) | Some concerns | Some concerns | Low | Low | Some concerns | Low | Some concerns | Prospective 10-year CLHLS cohort with repeated MMSE, extensive covariate adjustment and time-to-event/mixed modeling; lifestyle exposures were self-reported and substantial death/loss-to-follow-up occurred before the final analytic sample. |
| Shakersain et al. (2018) | Low | Some concerns | Low | Low | Some concerns | Low | Some concerns | Population-based longitudinal cohort with repeated MMSE and mixed-effects models; diet and leisure activities were self-reported and attrition over approximately 6 years may have introduced selection bias. |
| Dzierzewski et al. (2014) | Some concerns | Some concerns | Low | Some concerns | Some concerns | Low | Some concerns | Secondary longitudinal analysis of a lifestyle clinical trial using 18 weeks of daily diaries and multilevel models. Exercise and sleep were self-reported; this report estimates within- and between-person associations rather than a randomized intervention contrast. |

C. Randomized intervention studies (n = 6)

| **Study / tool** | **Randomization / allocation** | **Deviations / blinding** | **Outcome measurement** | **Missing data** | **Analysis / reporting** | **Overall** | **Main appraisal considerations** |
| --- | --- | --- | --- | --- | --- | --- | --- |
| Breneman et al. (2019) JBI RCT | Low / Unclear concealment | Some concerns | Low | Some concerns | Low | Some concerns | Participants were randomized to two walking doses and sleep was objectively assessed by actigraphy. Lack of a non-exercise control limits inference about exercise versus usual behavior; allocation concealment/blinding were not clearly reported. |
| Ghasemi and Cheraghbirjandi (2024) JBI RCT | Low / Unclear concealment | Some concerns | Some concerns | Unclear | Low | Some concerns | Randomized training/control design with objective metabolic outcomes, but allocation concealment, assessor blinding and attrition handling were not clearly documented in the accessible report; sleep was questionnaire-based. |
| Nagayama et al. (2021) JBI RCT | Low / Unclear concealment | Some concerns | Low | High risk | Some concerns | High risk | Computer-generated randomization was reported, but four post-randomization exclusions occurred from the intervention arm and the intended activity increase was small/variable. Final analyses were based on 11 intervention and 15 control participants rather than a full randomized sample. |
| Sewell et al. (2023) JBI RCT - secondary analysis | Low (parent trial) | Some concerns | Low | Low | Some concerns | Some concerns | Secondary moderator analysis of the IPAC randomized trial with intention-to-treat inclusion of participants with baseline sleep data. Sleep was self-reported and key moderation analyses excluded the control group, increasing selective-analysis concerns. |
| Walker et al. (2024) JBI RCT | Low / Unclear concealment | Some concerns | Low | Some concerns | Some concerns | Some concerns | Computer-generated randomization, objective exposure monitoring and laboratory outcomes were strengths. Five dropouts were excluded from analyses, adherence to the <2000-step target was low (41%), and secondary complier analyses may be vulnerable to post-randomization selection. |
| Maréchal et al. (2026) Cochrane RoB 2 crossover | Low | Some concerns | Low | Low | Low | Some concerns | Randomized three-condition crossover with standardized meals, repeated objective metabolic measurements and mixed models. Very small sample (n = 12) and limited information on washout/carryover procedures warrant caution for period/carryover bias. |

Interpretation and legend

Overall judgments: Low risk = no important risk-of-bias concern identified from the reported methods; Some concerns = at least one domain was unclear or plausibly vulnerable to bias without evidence of a major flaw; High risk = one or more methodological features were considered likely to materially affect the estimate. For JBI appraisals, these overall labels were derived qualitatively from domain-level judgments rather than from a numeric quality score.

Because the review is a scoping review, risk-of-bias findings were used to contextualize interpretation and were not used as exclusion criteria. For secondary analyses, appraisal focused on the analytic design and outcome used in this review rather than solely on the design of the parent study.

Appraisal frameworks

Barker TH, Hasanoff S, Aromataris E, Stone JC, Leonardi-Bee J, Sears K, et al. The revised JBI critical appraisal tool for the assessment of risk of bias for analytical cross-sectional studies. JBI Evidence Synthesis. 2026;24(3):401-408.

Barker TH, Hasanoff S, Aromataris E, Stone JC, Leonardi-Bee J, Sears K, et al. The revised JBI critical appraisal tool for the assessment of risk of bias for cohort studies. JBI Evidence Synthesis. 2025;23(3):441-453.

Barker TH, Stone JC, Sears K, Klugar M, Tufanaru C, Leonardi-Bee J, Aromataris E, Munn Z. The revised JBI critical appraisal tool for the assessment of risk of bias for randomized controlled trials. JBI Evidence Synthesis. 2023;21(3):494-506.

Cochrane Risk of Bias 2 (RoB 2) tool for crossover trials, revised 18 March 2021.
